# Research team diversity impacts scientific output in allergy and immunology programs^☆^

**DOI:** 10.1016/j.waojou.2024.101004

**Published:** 2024-12-12

**Authors:** Takeya Adachi,, Norika Narimatsu, Yasushi Ogawa,, Masako Toriya,, Tamami Fukushi,, Masashi Shirabe,, Masaki Futamura,, Takenori Inomata,, Keigo Kainuma,, Keiko Kan-o,, Yosuke Kurashima,, Katsunori Masaki,, Saeko Nakajima,, Masafumi Sakashita,, Sakura Sato,, Mayumi Tamari,, Hideaki Morita,, Amane Koizumi,

**Affiliations:** a Department of Dermatology, Keio University School of Medicine, Tokyo, Japan; b Allergy Center, Keio University Hospital, Tokyo, Japan; c Keio Frontier Research & Education Collaborative Square (K-FRECS) at Tonomachi, Keio University, Kanagawa, Japan; d Department of Medical Innovation and Translational Medical Science, Graduate School of Medical Science, Kyoto Prefectural University of Medicine, Kyoto, Japan; e ENGAGE Task Force, Tokyo, Japan; f Department of Computer Science, Leiden University, Leiden, Netherlands; g Department of Advanced Medicine, Department of Dermatology, Nagoya University Hospital, Aichi, Japan; h Global Research Institute, Keio University, Tokyo, Japan; i Tokyo Online University, Tokyo, Japan; j Institute of Science Tokyo, Tokyo, Japan; k Department of Pediatrics, NHO Nagoya Medical Center, Aichi, Japan; l Department of Ophthalmology, Juntendo University Graduate School of Medicine, Tokyo, Japan; m Department of Hospital Administration, Juntendo University Graduate School of Medicine, Tokyo, Japan; n AI Incubation Farm, Juntendo University Graduate School of Medicine, Tokyo, Japan; o Department of Telemedicine and Mobile Health, Juntendo University Graduate School of Medicine, Tokyo, Japan; p Institute for Clinical Research, NHO Mie National Hospital, Mie, Japan; q Department of Respiratory Medicine, Tokyo Women's Medical University, Tokyo, Japan; r Department of Mucosal Immunology, Graduate School of Medicine, Chiba University, Chiba, Japan; s Division of Pulmonary Medicine, Department of Medicine, Keio University School of Medicine, Tokyo, Japan; t Department of Drug Discovery for Inflammatory Skin Diseases, Kyoto University Graduate School of Medicine, Kyoto, Japan; u Department of Otorhinolaryngology Head and Neck Surgery, University of Fukui, Fukui, Japan; v Department of Allergy, Clinical Research Center for Allergy and Rheumatology, NHO Sagamihara National Hospital, Kanagawa, Japan; w Division of Molecular Genetics, The Jikei University School of Medicine, Research Center for Medical Science, Tokyo, Japan; x Department of Allergy and Clinical Immunology, National Research Institute for Child Health and Development, Tokyo, Japan; y Allergy Center, National Center for Child Health and Development, Tokyo, Japan; z National Institutes of Natural Science, Tokyo, Japan

**Keywords:** Allergy, Impact analysis, Interdisciplinary research, Research output, Research team diversity

## Abstract

**Background:**

This study examined the relationship between the disciplinary diversity of research teams and research output (RO) in allergy and immunology programs funded by the National Institutes of Health (NIH) in the United States, Medical Research Council (MRC) in the United Kingdom, and Japan Society for the Promotion of Science (JSPS).

**Methods:**

Using a dataset containing 1243, 3645, and 1468 articles funded by the NIH, MRC, and JSPS, respectively, we analyzed the correlation between disciplinary diversity and RO in allergy and immunology programs that received grants from 2017 to 2021. Diversity was measured using All Science Journal Classification codes counts, Shannon-Wiener index, and newly developed Omnidisciplinary index (o-index). The impact of diversity on RO was evaluated Normalized Paper Count (reflecting research quantity), Normalized Top 1% Paper Count (reflecting research excellence), and Normalized Top 10% Paper Count (reflecting research substantiality).

**Results:**

There were no significant differences in diversity between the funding agencies, indicating a marginal relationship between team composition and RO (p = 0.641 for Shannon-Winner index). RO was positively correlated with team diversity in NIH- and MRC-funded programs and positively correlated with the degree of specialization in JSPS-funded programs.

**Conclusions:**

These results underscore the complexity of the relationship between research team diversity and RO and the influence of contextual factors such as country-specific characteristics and grant program objectives. Specifically, the analysis of JSPS-funded groups suggests that the degree of specialization has a greater impact on RO than disciplinary diversity. This study contributes to ongoing efforts to optimize team composition to improve RO in allergy and immunology programs.

## Graphical abstract

**Figure:**
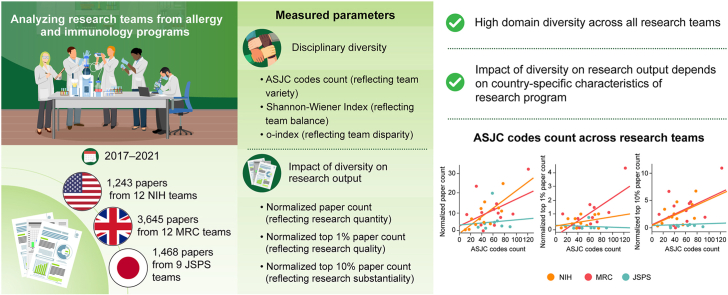

## Introduction

Allergic and immunological diseases significantly jeopardize public health, affecting multiple organ systems and a large percentage of the population and manifesting symptoms from childhood to adulthood.^1^ The exposure to allergens increases with age and number of outdoor activities.^2^ Furthermore, some allergic conditions occur across geographies and persist in several regions, even during pandemics.^3^

In this context, advancements in treatment and research and development (R&D) in allergy and immunology require a comprehensive and interdisciplinary approach.^4^ Research collaboration is imperative and should extend beyond academia and pharmaceutical companies and include the food, clothing, and housing industries to treat complex multifaceted diseases more effectively.^5^^,^^6^

Public funding is pivotal in fostering R&D.^7^ However, the optimal allocation of limited resources necessitates the recognition of the unique characteristics and expectations of public funds, which differ markedly from industry funding.^8^ Public funds support long-term research, which may be overlooked by other funding sources because of limited returns. Moreover, the outcomes of publicly funded R&D are expected to benefit taxpayers and society.

Research output (RO) is traditionally evaluated using quantitative metrics such as the number of academic papers published, intellectual property, and journal impact factor.^9^ In turn, qualitative evaluations such as the field-weighted citation impact (FWCI) are increasingly used but focus on short-term indicators.^10^

To identify effective performance indicators that shape the allocation of research funds, we previously assessed the impact of research supported by funding agencies (FAs) in the United Kingdom, the United States, and Japan.^11^ This multifaceted analysis evaluated long-term indicators of research substantiality^12^ and employed natural language analysis to assess contributions to allergy and immunology programs in Japan.^6^^,^^13^ Our findings underscored the variability in RO across stages and disciplines. This variability is strongly influenced by country-specific characteristics and FA objectives. The results highlight the need for an assessment approach tailored to the unique characteristics, strengths, weaknesses, and potential of each country's research ecosystem, thereby enabling a more effective and targeted funding strategy.

To assess the current landscape of national and global R&D, we followed up on previous studies and assessed the role of disciplinary diversity on the RO of research teams in the allergy and immunology funded by FAs from 3 different regions: Japan Society for the Promotion of Science (JSPS) from Asia, National Institutes of Health (NIH) from the United States, and Medical Research Council (MRC) from the United Kingdom. We employ a diverse array of metrics to scrutinize and compare the composition of these research teams. Furthermore, our study seeks to elucidate the intricate relationship between the diversity of research teams and their research outputs, thereby highlighting the pivotal role team diversity plays in enhancing the impact of research.

## Methods

### Selection of research projects

**Fig. 1:**
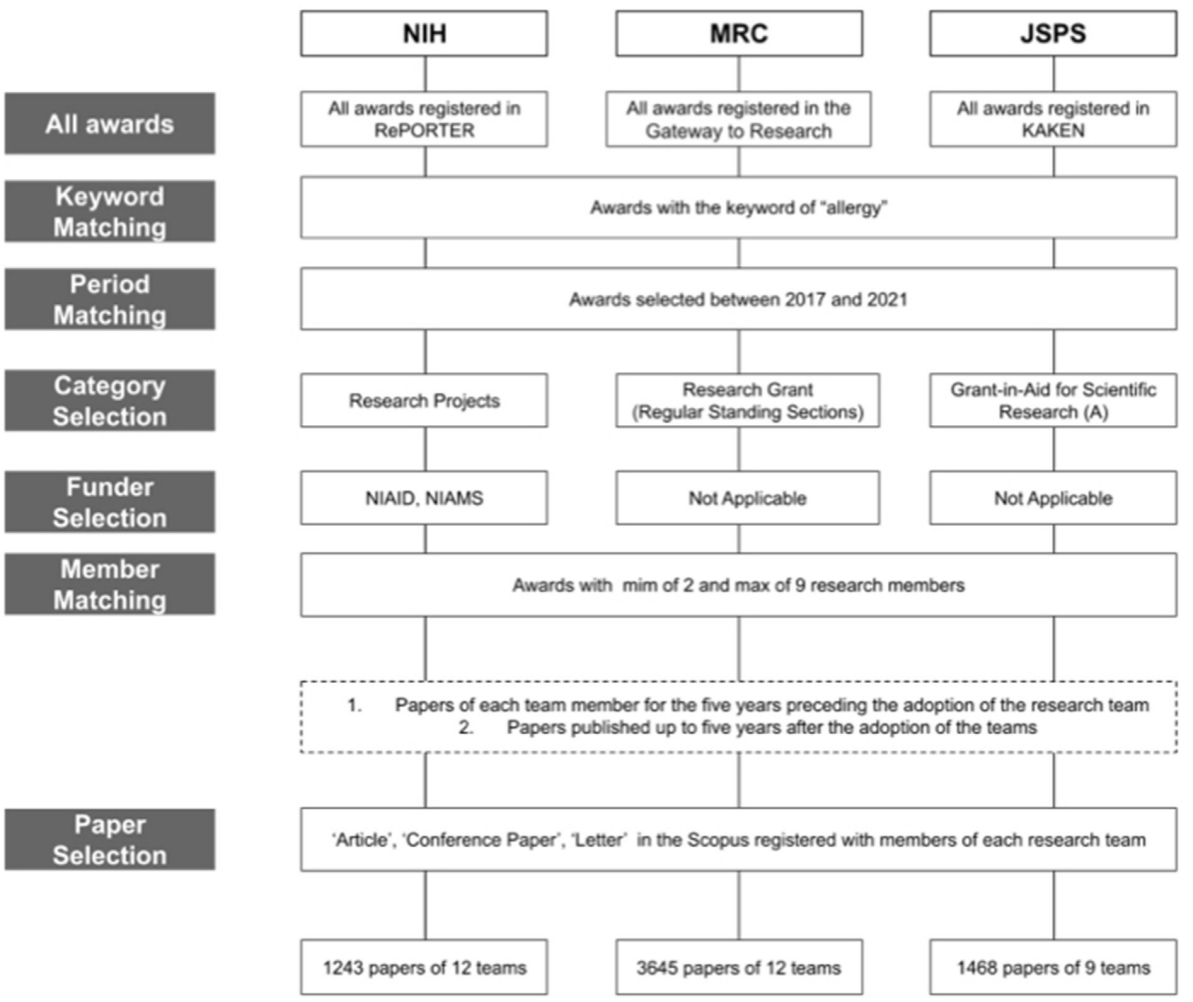
**Selection of research projects.** Research articles were selected from the National Institutes of Health (NIH), Medical Research Council (MRC), and Japan Society for the Promotion of Science (JSPS) databases by keyword matching, period alignment, category selection, funder identification, and team member matching. A total of 1243 papers from 12 NIH teams, 3645 articles from 12 MRC teams, and 1468 papers from 9 JSPS teams were selected

This research was conducted under the approval by the National Institute of Natural Science (Approval Code 100053271). Data on research teams were extracted from the NIH, MRC, and JSPS databases, including RePORTER,^14^ UKRI Gateway,^15^ and KAKEN, respectively^16^ (Fig. 1). In each database, research projects funded by these FAs between 2017 and 2021 and whose titles or summaries included the keyword “allergy” were identified and selected. To standardize the scope of the study, we selected projects from specific categories, including awards granted by the National Institute of Allergy and Infectious Diseases and National Institute of Arthritis and Musculoskeletal and Skin Diseases (NIH), Research Grants from Regular Standing Sections (MRC), and the Grant-in-Aid for Scientific Research (A) (KAKEN). Teams with 2–9 members were included in the study.

Data from articles, conference papers, and letters were extracted from Scopus^17^ to assess the RO of each researcher from the 5-year period before and the 5-year period after team formation. Data from the 5 years before team formation were used to evaluate disciplinary diversity of research teams, while data from the 5 years after were used to measure RO. This data collection strategy allowed the comprehensive evaluation of disciplinary diversity and RO.

### Measurement of team diversity

**Supplementary Figure 1:**
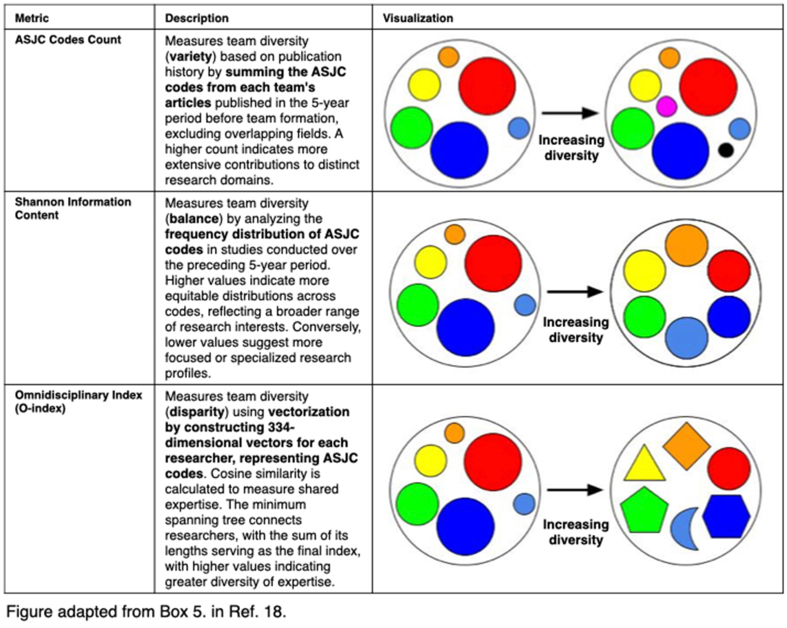
Schema of team diversity indices.Metrics to measure disciplinary diversity based on three dimensions: variety (how many fields it consists of), balance (how much it consists of), and disparity (how many different fields it consists of).

There are a number of indicators to measure disciplinary diversity, and the most common methods are based on 3 dimensions: variety (the number of different fields involved), balance (the relative representation of each field), and disparity (the degree of difference between the fields) (Supplementary Fig. 1).^18^ In our analysis, we evaluated team diversity with the following indices.

1. All Science Journal Classification (ASJC) codes count: This index measures team diversity (variety) based on publication history.^19^ We collected information on articles published in the 5-year period before team formation. We used the ASJC system from Scopus as a standardized classification scheme and summed the counts for each team. Overlapping fields were excluded to ensure a more accurate reflection of distinct research domains. A higher count indicates that the team comprises individuals with a history of contributing to various research domains.
2. Shannon information content (SIC):^20^ This index measures diversity (balance) by calculating the frequency distribution of each ASJC code in studies conducted over the preceding 5-year period before team formation. Diversity is higher when the frequencies of ASJC codes are more equitable, indicating a broader range of research interests. Conversely, diversity is lower when frequencies are less equitable, indicating a more focused or specialized research profile in the team.
3. Omnidisciplinary index (o-index): This metric evaluates diversity (disparity) through vectorization. For each researcher, a 334-dimension vector was constructed, with each vector representing an ASJC code. We calculated the cosine similarity for each research pair, with 0 indicating no shared codes and 1 indicating an identical vector orientation. To quantify the degree of separation between researchers, we subtracted the cosine similarity from 1. The unique aspect of this metric lies in its reliance on vectors that capture individual contributions and relationships among researchers in each team. The minimum spanning tree is employed to connect all researchers with the shortest edge weights, and the sum of the lengths of the sides of the minimum spanning tree serves as the final index, offering a numerical representation of the diversity of expertise.^21^ A higher value indicates a higher diversity of expertise.

These 3 indexes were computed based on the ASJC codes of published papers, with the fundamental assumption that expertise is faithfully represented in published work. This presupposition implies that the topics addressed in publications are dependable indicators of the diverse fields in which they have actively contributed to knowledge and research. We also assumed that each field had an equal relative importance or significance assigned to it. These metrics do not distinguish between the significance or depth of individual contributions in each ASJC code and treat all fields as equal contributors to overall team diversity.

### RO measurement

RO was assessed using a multifaceted approach involving quantitative, qualitative, and substantial indexes.

1. Normalized Paper Count (NPC): The RO of each team was quantified by counting the total number of papers published after team formation.
2. Normalized Top 1% paper Count: The excellence and impact of RO were evaluated by counting the number of papers that fall within the Top 1% of their respective fields based on citation metriscs.^22^^,^^23^
3. Normalized Top 10% Paper Count: The long-term contributions of RO were assessed by counting the number of papers that fall within the Top 10% of their respective fields, institutional h-5 index, and disruption percentile.^12^^,^^24^^,^^25^ In this study, we chose to focus on the Top 10% of Papers Count. This metric is recognized as a reliable indicator of long-term contributions, reflecting the research output's consistent prominence and influence within the field, as reported by Shirabe et al.^12^

The values for each indicator were normalized by dividing the total number of papers by the number of years since publication and the number of team members.

### Statistical analyses

Patterns of diversity were evaluated by analysis of variance (ANOVA). ANOVA was selected for its robustness in accommodating normal distribution assumptions and ensuring the homogeneity of variances, which are essential prerequisites for comparative analyses. The assumptions of normality and homogeneity of variances were substantiated using the Shapiro-Wilk test and Bartlett test, respectively. Kruskal-Wallis test was used for the data that did not meet the normality assumption. The relationship between team diversity and RO was analyzed using Pearson's correlation coefficient (R).

## Results

This study focused on allergy and immunology programs funded by the NIH, MRC, and JSPS between 2017 and 2021. The analysis included 1243 papers from 12 NIH teams, 3645 papers from 12 MRC teams, and 1468 papers from 9 JSPS teams (Fig. 1). While there was a notable concentration of NIH groups in 2020, and the dataset did not include the MRC team from 2017 and the JSPS team from 2018, the distribution of the start years for the included teams was balanced (Table 1).

**Table 1:** Characteristics of research groups enrolled in allergy and immunology programs funded by the National Institutes of Health, Medical Research Council, and Japan Society for the Promotion of Science

	NIH	MRC	JSPS
Team count	12	12	9
2017	3	0	2
2018	1	1	0
2019	1	5	2
2020	6	3	3
2021	1	3	2
Avg. Normalized citation count	10.1	6.00	1.55
Avg. Normalized paper count	2.59	3.30	2.08
Avg. FWCI per team	3.57	3.59	1.08
Avg. Researchers per team	2.25	4.83	5.11
Avg. Grant Amount	63.5millionJPY	61.6millionJPY	43.2millionJPY

The characteristics and performance of research teams across FAs are shown in Table 1. The results showed that the NIH teams had more citations per paper, and the MRC teams had a higher number of papers. Moreover, the NIH teams tended to have fewer members, and the JSPS teams received slightly fewer grants.

**Fig. 2:**
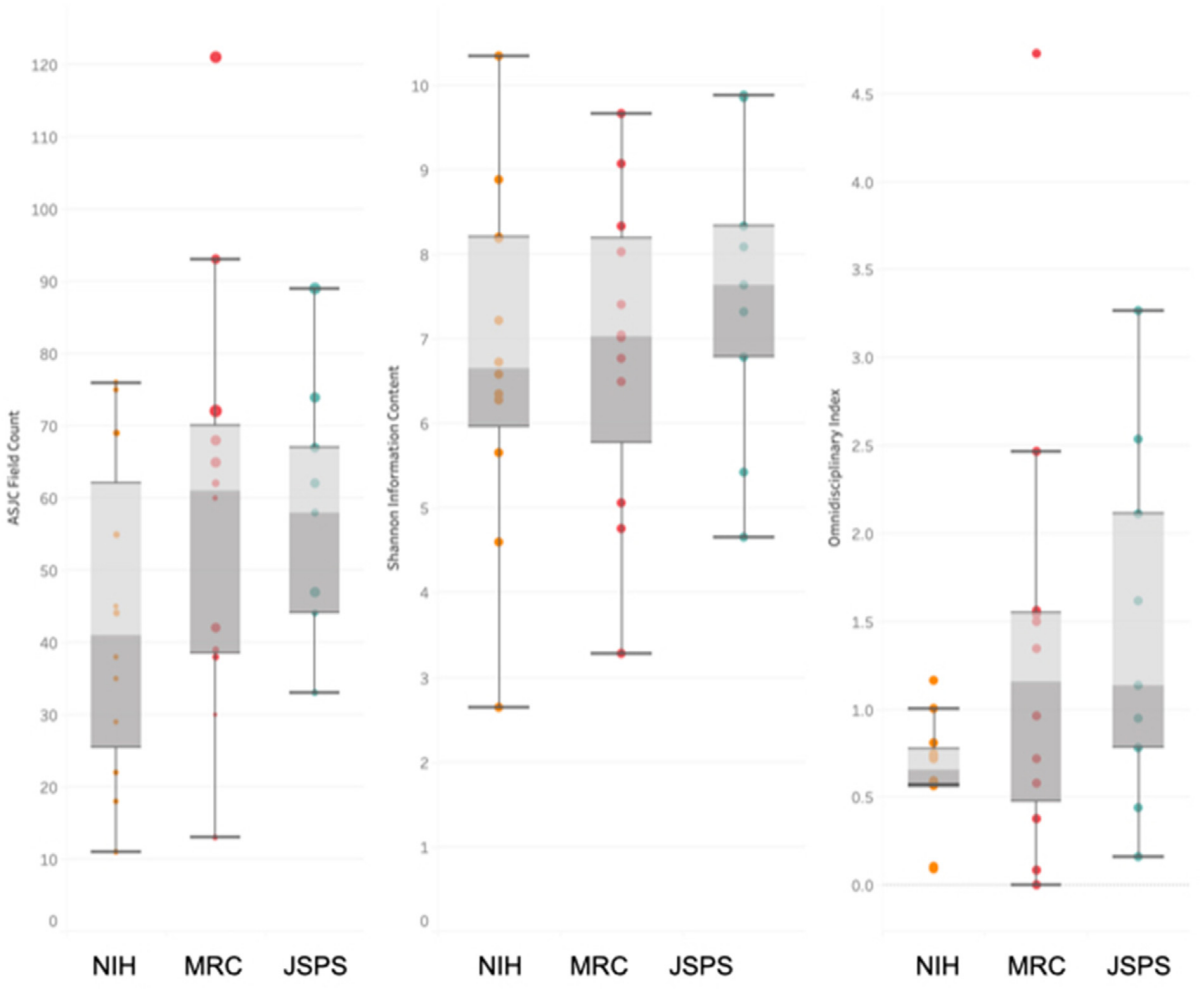
**Diversity of research groups funded by3 funding agencies.** The diversity of research groups from allergy and immunology programs funded by the National Institutes of Health, Medical Research Council, and Japan Society for the Promotion of Science was assessed by measuring the ASJC codes count (variety), Shannon information content (balance), and Omnidisciplinary index (disparity). Each index provides a distinct perspective on the compositional diversity of research teams funded by these 3 agencies

The analysis of the diversity of the research groups supported by the 3 FAs is shown in Fig. 2 and Table 2. Diversity was slightly lower in the NIH group. The ASJC codes count and SIC data had normal distributions and variance homogeneity, confirmed by the Shapiro-Wilk test and Bartlett test, respectively. However, one-way ANOVA indicated that the differences in diversity indices were similar between the groups. The data on the o-index did not meet the normality assumption and thus were analyzed using the Kruskal-Wallis test. There were no significant between-group differences in this index.

**Table 2:** Diversity of research groups enrolled in allergy and immunology programs funded by the National Institutes of Health, Medical Research Council, and Japan Society for the Promotion of Science

ASJC Field Counts			
	NIH	MRC	JSPS
Mean	43.1	58.6	56.3
Std	22.9	29.2	18.6
Shapiro-Wilk	All group follow a normal distribution		
Bartlette test	p-value: 0.401		
One-way ANOVA	p-value: 0.262		

**ASJC field counts**			
	**NIH**	**MRC**	**JSPS**

Mean	6.81	6.91	7.56
Std	2.02	1.84	1.77
Shapiro-Wilk	All group follow a normal distribution		
Bartlette test	p-value: 0.917		
One-way ANOVA	p-value: 0.641		

**Omnidisciplinary index**			
	**NIH**	**MRC**	**JSPS**

Mean	0.641	1.32	1.44
Std	0.311	1.29	1.03
Shapiro-Wilk	Data did not meet the assumption of normality		
Kruskal-Wallis	p-value: 0.135		

**Fig. 3:**
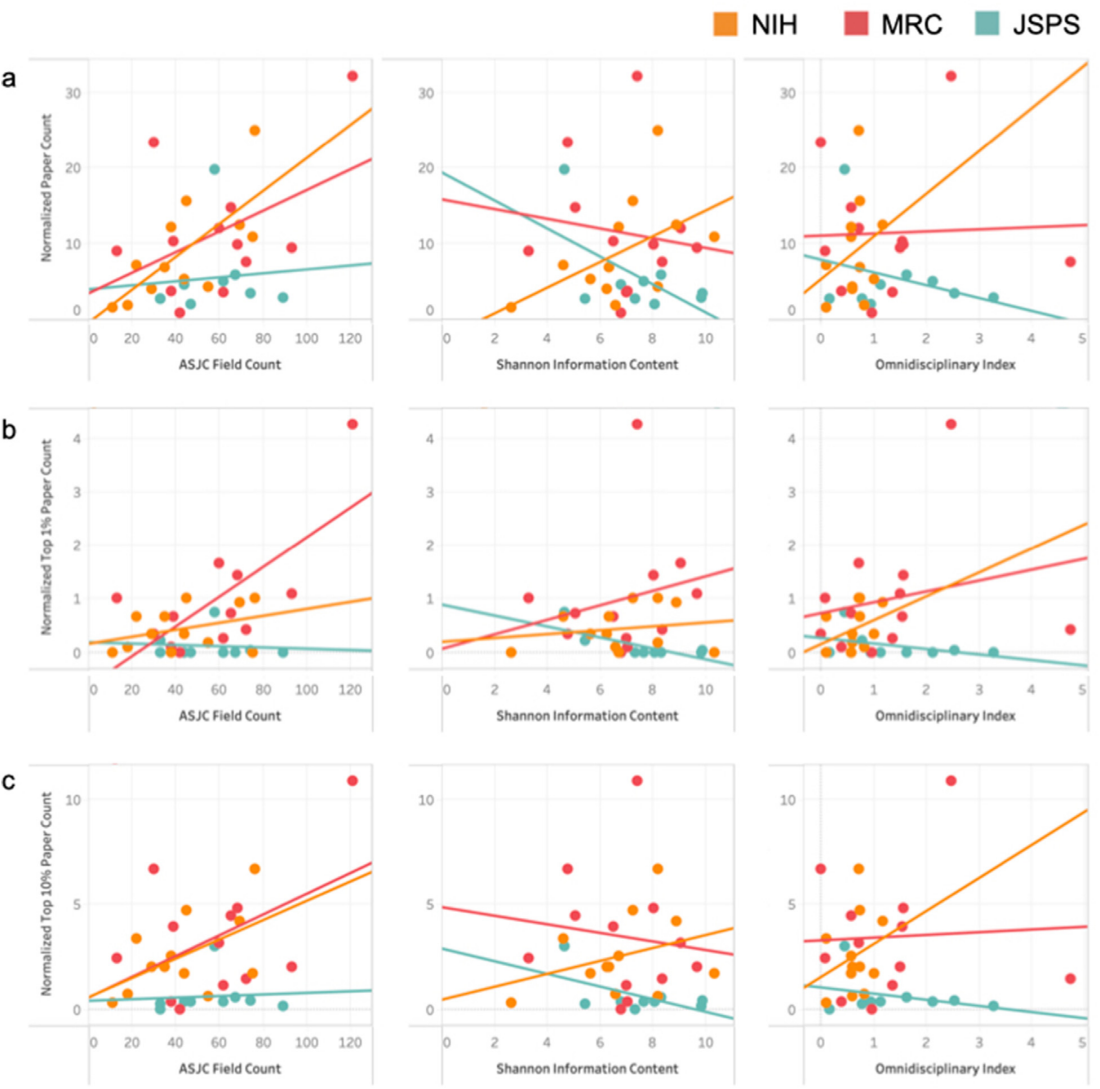
**Correlation between team diversity and research output across funding agencies.** Correlations between 3 measures of team diversity—ASJC codes count (variety), Shannon information content (balance), and Omnidisciplinary index (disparity)—and 3 dimensions of research output—normalized paper count (quantity), normalized top 1% paper count (excellence), and normalized top 10% paper count (substantiality) in research programs funded by the National Institutes of Health, Medical Research Council, and Japan Society for the Promotion of Science

The relationship between team diversity and RO was further assessed by calculating the correlation between 3 diversity indices and 3 measures of RO: Normalized Paper Count (reflecting research quantity), Normalized Top 1% Paper Count (reflecting research excellence), and Normalized Top 10% Paper Count (reflecting research substantiality), (Fig. 3 and Table 3). There was a significant positive correlation between the ASJC codes count and NPC in NIH-funded groups (slope = 0.217; R^2^ = 0.500, *P* = 0.010), suggesting that higher team variety increased RO. Similarly, there was a positive association between the ASJC codes count and Normalized Top 1% Paper Count in NIH-funded teams (slope = 0.028, R^2^ = 0.495, *P* = 0.011), indicating that higher variety improved the excellence of RO.

**Table 3:** Correlation analysis between team diversity indices and research outputs in programs funded by the National Institutes of Health, Medical Research Council, and Japan Society for the Promotion of Science

ASJC Field Count	FA	$R^{2}$	p-value	Condition	Value	SE
Normalized paper count	NIH	0.500	0.010	Slope	0.217	0.069
				Intercept	−0.436	3.29
Normalized paper count	MRC	0.208	0.136	Slope	0.136	0.084
				Intercept	3.41	5.46
Normalized paper count	JSPS	0.008	0.818	Slope	0.026	0.110
				Intercept	3.95	6.49
Normalized Top1% paper count	NIH	0.128	0.253	Slope	0.007	0.005
				Intercept	0.152	0.258
Normalized Top1% paper count	MRC	0.495	0.011	Slope	0.028	0.009
				Intercept	−0.635	0.576
Normalized Top1% paper count	JSPS	0.008	0.817	Slope	−0.001	0.005
				Intercept	0.179	0.295
Normalized Top10% paper count	NIH	0.285	0.074	Slope	0.046	0.023
				Intercept	0.544	1.10
Normalized Top10% paper count	MRC	0.224	0.120	Slope	0.049	0.029
				Intercept	0.532	1.89
Normalized Top10% paper count	JSPS	0.006	0.843	Slope	0.004	0.018
				Intercept	0.378	1.08
**Shannon informational content**	FA	$R^{2}$	p-value	Condition	Value	SE

Normalized paper count	NIH	0.258	0.092	Slope	1.70	0.909
				Intercept	−2.62	6.43
Normalized paper count	MRC	0.018	0.677	Slope	−0.639	1.49
				Intercept	15.8	10.6
Normalized paper count	JSPS	0.352	0.092	Slope	−1.85	0.946
				Intercept	19.4	7.32
Normalized Top1% paper count	NIH	0.033	0.571	Slope	0.036	0.062
			Intercept	0.187	0.436	
Normalized Top1% paper count	MRC	0.047	0.499	Slope	0.136	0.193
				Intercept	0.056	1.38
Normalized Top1% paper count	JSPS	0.526	0.027	Slope	−0.102	0.037
				Intercept	0.885	0.284
Normalized Top10% paper count	NIH	0.108	0.296	Slope	0.308	0.280
				Intercept	0.428	1.98
Normalized Top10% paper count	MRC	0.015	0.703	Slope	−0.204	0.521
				Intercept	4.84	3.72
Normalized Top10% paper count	JSPS	0.340	0.099	Slope	−0.302	0.159
				Intercept	2.87	1.23
**Omnidisciplinary index**	FA	$R^{2}$	p-value	Condition	Value	SE

Normalized paper count	NIH	0.068	0.414	Slope	5.63	6.61
				Intercept	5.31	4.68
Normalized paper count	MRC	0.002	0.900	Slope	0.276	2.14
				Intercept	11.0	3.87
Normalized paper count	JSPS	0.099	0.410	Slope	−1.69	1.93
				Intercept	7.87	3.35
Normalized Top1% paper count	NIH	0.121	0.268	Slope	0.447	0.381
				Intercept	0.260	0.145
Normalized Top1% paper count	MRC	0.052	0.476	Slope	0.204	0.275
				Intercept	0.725	0.498
Normalized Top1% paper count	JSPS	0.178	0.258	Slope	−0.103	0.084
				Intercept	0.146	0.270
Normalized Top10% paper count	NIH	0.067	0.417	Slope	1.57	1.86
				Intercept	1.52	1.31
Normalized Top10% paper count	MRC	0.003	0.866	Slope	0.129	0.748
				Intercept	3.26	1.35
Normalized Top10% paper count	JSPS	0.107	0.390	Slope	−0.293	0.319
				Intercept	1.01	0.555

There was a positive but not significant correlation between the SIC and NPC in the NIH group (slope = 1.70, R^2^ = 0.258, *P* = 0.092) and a negative but not significant correlation between the SIC and NPC in the JSPS group (slope = −1.85, R^2^ = 0.352, *P* = 0.092), suggesting that higher diversity is not always associated with a higher quantity of RO. In the MRC group, there was a significant negative correlation between the Normalized Top 1% paper count and the SIC (slope = −0.102, R^2^ = 0.526, *P* = 0.027), demonstrating the complex relationship between diversity and high-impact RO.

There was no significant correlation between diversity and o-index. Nonetheless, the non-significant correlation between the ASJC codes count and o-index illustrates the nuanced dynamics of team diversity and its impact on RO.

## Discussion

With advancements in scientific knowledge, the advantages of diversity and specialization in research teams have become multidimensional. Numerous studies assessed the effects of gender, race, and academia-industry collaborations on RO.^26^, ^27^, ^28^ The present study scrutinized the effect of team diversity on RO in allergy and immunology programs. The results demonstrate the nuanced impact of disciplinary diversity on RO, which varies significantly across programs. Notably, RO was positively associated with team diversity in NIH- and MRC-funded programs and positively correlated with the degree of specialization of the teams in JSPS-funded programs. The distinctions between the NIH, MRC, and JSPS underscore the need to acknowledge the distinctive characteristics and objectives of each FA.

**Supplementary Figure 2:**
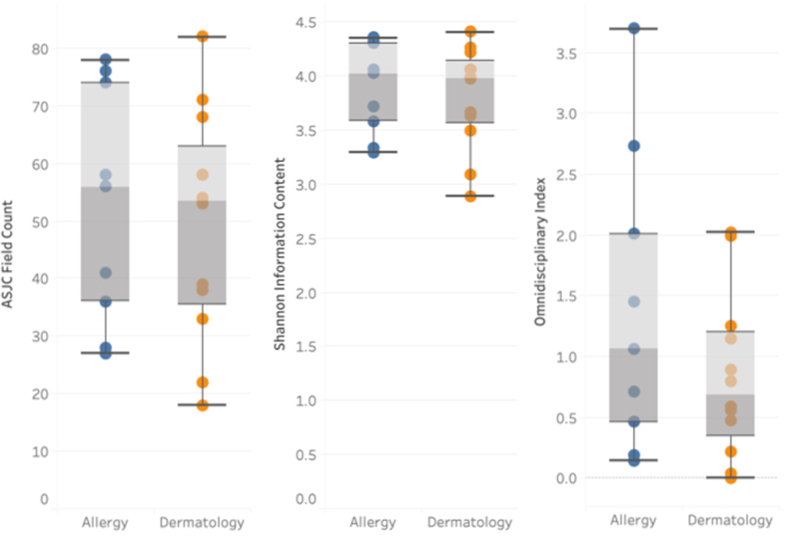
Diversity indices of Allergy and Dermatology research teams funded by JSPS research program. Comparison of ASJC codes count (variety), Shannon information content (balance), and Omnidisciplinary index (disparity) ASJC between Allergy and Dermatology research teams funded by Grant-in-Aid for Scientific Research on Innovative Areas (KAKENHI A) of JSPS.

This study evaluated allergy and immunology programs to account for the complex nature of allergic diseases that affect multiple organ systems in all life stages, jeopardizing public health in developing and developed countries and requiring multidisciplinary R&D approaches. We found no significant differences in diversity (variety, balance, and disparity) between groups funded by the NIH, MRC, and JSPS. ASJC code counts were consistently above 40, underscoring the critical role of interdisciplinarity in advancing allergy research worldwide. In fact, when we measured the diversity indices of 12 research teams in the field of dermatology, which is considered to cover mores specialized areas in the Grant-in-Aid for Scientific Research on Innovative Areas (KAKENHI A) from 2017 to 2021, we found a similar or slightly lower trend than in the field of allergy (Supplementary Fig. 2). Furthermore, The Grants-in-Aid for Scientific Research provided by the JSPS cater to different objectives. While the KAKENHI A enrolled in this study emphasizes “creative/pioneering research conducted by 1 researcher or multiple researchers” without advocating for diversity or interdisciplinary collaboration, the “Grant-in-Aid for Scientific Research on Innovative Areas” proposed the creation of novel research areas, and the “Grant-in-Aid for Transformative Research Areas” capitalizes on interdisciplinary research.^29^ Therefore, it is possible that projects under other JSPS themes subsidize more interdisciplinary research teams than KAKENHI A.

Our findings also indicate that the interpretation of the results varies depending on the definition of team diversity. The positive correlation between RO and the number of ASJC codes and the absence of correlation between RO and o-index (reflecting disparity) in the NIH and MRC groups underscore the importance of team variety. This suggests that the inclusion of members from diverse disciplinary backgrounds might be more crucial than simply having a mix of heterogeneous researchers in this case. Conversely, the negative correlation between the RO and SIC and absence of a correlation between the RO and o-index in the JSPS group imply that the benefits of diversity may require teams to be composed of members with equally deep expertise in their respective fields rather than broadening the overall expertise of the team. Thus, these indicators should be evaluated jointly to better understand the effect of team diversity on RO.

**Supplementary Figure 3:**
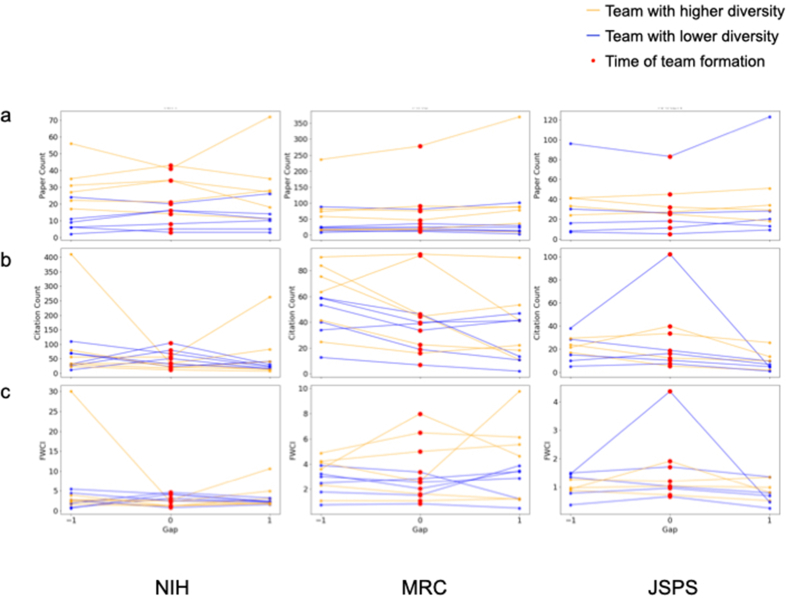
Annual trends in research output from the 1-year period before to the 1-year period after team formation (red circle). Research output was assessed by measuring the (a) number of published articles, (b) number of citations per article, and (c) field-weighted citation impact. The teams funded by three agencies—National Institutes of Health, Medical Research Council, and Japan Society for the Promotion of Science—were categorized into two groups based on the degree of diversity (high and low).

This study has limitations. First, although we evaluated the correlation between team diversity and RO, the analysis of causal relationships was beyond the scope of the study. Consequently, we cannot explain how team diversity (independent variable) influences RO (dependent variable) and why the correlation between these variables differs across funding programs. We also observed an upward trend in research outputs for teams with higher diversity at NIH and MRC, and for teams with higher expertise (lower diversity) at JSPS, yet these did not reach statistical significance (Supplementary Fig. 3). Future studies could benefit from incorporating mediation analysis to unearth potential intermediary correlation factors.^30^ Third, this analysis primarily quantified team diversity by focusing on the number of fields of researchers. Investigating other dimensions of diversity−such as researchers' affiliations, titles, nationalities, and genders−could offer a more comprehensive understanding.

This study examines the relationship between disciplinary diversity and RO in allergy and immunology programs. Diversity was similar between groups funded by different FAs, highlighting the complex interplay between team composition and RO. In turn, the findings suggest a contextual dependence on the country and specific objectives and characteristics of funding programs. As the complexity of scientific and medical challenges escalates, further research is essential to explore how diversity or specialty within research teams may enhance overall research capacity and contribute to scientific and technological innovation strategy across various fields.

## Authors’ consent for publication

All the authors have approved the submission of this manuscript.

## Availability of data and materials

All the data included in this analysis were extracted from the following open database.

- National Institutes of Health. *RePORTER.* Available at: https://reporter.nih.gov, Accessed January 23, 2024.

- *UK Research and Innovation: UKRI Gateway.* Available at: https://gtr.ukri.org, Accessed January 23, 2024.

- Japan Society for the Promotion of Science. KAKEN database. Available at: https://kaken.nii.ac.jp/en/, Accessed January 23, 2024.

## Authors’ contributions

TA, NN, YO, MTo, TF, MS, and AK designed the study. NN, YO, and MTo analyzed data. TA, TF, MSh, and AK obtained and analyzed data. MF, TI, KKai, KKan, YK, KM, SN, MSa, SS, MTa, and HM interpreted data and critically revised the manuscript for important intellectual content. TA and NN drafted the manuscript. TA and AK managed the study and critically revised the manuscript for important intellectual content. All authors have read and approved the final version of the manuscript.

## Ethics approval

The research components of this platform were conducted under the approval by the National Institutes of Natural Science (Approval Code 100053271).

## Funding

This research was supported by the Scientific Research Fund of the Ministry of Health, Labour and Welfare, Japan (Grant Number 21FE2001), AMED (Grant Number 23ek0410090), JSPS KAKENHI (Grant Number: 22K16268), JST/RISTEX “Science, Technology, and Innovation Policy” Research Program, and Research Project Keio 2040 (Creativity Initiative) at Keio University Global Research Institute.

## Declaration of competing interest

The authors have no actual or potential conflicts of interest to declare.
